# Caspase 12 degrades IκBα protein and enhances MMP-9 expression in human nasopharyngeal carcinoma cell invasion

**DOI:** 10.18632/oncotarget.16535

**Published:** 2017-03-23

**Authors:** Wing-Keung Chu, Chih-Chin Hsu, Shiang-Fu Huang, Chia-Chi Hsu, Shu-Er Chow

**Affiliations:** ^1^ Department of Physiology, Chang Gung University, Taoyuan, Taiwan; ^2^ Center for Healthy and Aging Research, College of Medicine, Chang Gung University, Taoyuan, Taiwan; ^3^ Department of Physical Medicine and Rehabilitation, Chang Gung Memorial Hospital at Keelung, Keelung, Taiwan; ^4^ Department of Traditional Chinese Medicine, College of Medicine, Chang Gung University, Taoyuan, Taiwan; ^5^ Department of Otolaryngology, Head and Neck Surgery, Chang Gung Memorial Hospital, Taoyuan, Taiwan; ^6^ Department of Nature Science, Center for General Studies, Chang Gung University, Taoyuan, Taiwan

**Keywords:** Caspase-12, cell invasion, IκBα, NF-κB, human nasopharyngeal carcinoma

## Abstract

Caspase-12 (Casp12), an inflammatory caspase, functions as a dominant-negative regulator of inflammatory responses and is associated with the signaling of apoptosis. However, the physiological function of Casp12 presented in cancer cells is still unclear. This study demonstrated that overexpression of Casp12 mediated IκBα degradation and significantly increased NF-κB activity. Exposure of human nasopharyngeal carcinoma (NPC) cells to phorbol-12-myristate-13-acetate (PMA) increased the levels of Casp12 and MMP-9 resulting in NPC cell invasion. Target suppression of Casp12 by small interfering RNA (siRNA) or an inhibitor of Casp12 markedly decreased the level of PMA-induced MMP-9 protein and cell invasion. Moreover, suppression of Casp12 significantly inhibited the basal activity of NF-κB and decreased the PMA-induced NF-κB reporter activity. The effect of Casp12 on NF-κB activation was indicated via the post-translational degradation of IκB. This study revealed that a critical role of Casp12 on the activation of NF-κB via IκBα degradation which provides a link between inflammatory and aggressive invasion in NPC cells.

## INTRODUCTION

Human nasopharyngeal carcinoma (NPC) is one of the most predominant head and neck cancer in southern China and South East Asia, the Arctic, and the Middle East/North Africa [1]. Developing NPC is associated with a multiple risk factors which include elevated antibody titers against the Epstein-Barr virus (EBV), consumption of salt-preserved fish, a family history of NPC, and certain human leukocyte antigen class I genotypes [1]. EBV has been suggested to have an important role in the pathogenesis and development of NPC. EBV encoded viral latent membrane protein 1 (LMP1) has been to display oncogenic properties in rodent fibroblasts and induces profound morphological and phenotypic effects in epithelial cells. However, EBV infection alone is not a sufficient cause of NPC, because virtually all adults worldwide are infected with the virus, yet only a small proportion of individuals develop NPC [1]. Many of NPC cell lines do not expression EBV transcripts in long-term culture [2]. Apparently, the possible role of EBV in the pathogenesis of NPC is still controversial. The distinctive racial/ethnic and geographic distribution of NPC worldwide suggests that both environmental factors and genetic traits contribute to its development [3]. Sinonasal tract inflammation is associated with NPC, suggesting an important link of the pro-inflammatory factors for the carcinogenesis of NPC [4, 5]. Inflammation has a driving force expediting cancer metastasis [6, 7]. It is likely that inflammatory caspases (caspase-1, -4, -5, -12) are important mediators of the innate immune response and activated by the inflammasome and facilitate the activation and secretion of inflammatory cytokines (1). Among of these, Caspase 12 (Casp12) occurs in individuals of African descent and carriers has endotoxin hypo-responsiveness and an increased susceptibility to severe sepsis [8]. It functions as a dominant-negative regulator that dampens Casp1 activation and inhibits secretion of interleukine-1β (IL-1β) and IL-18 [8, 9]. Interestingly, a recent study indicates Casp12 deficiency fails to increase Casp1 activation and IL-1β and IL-18 releasing [10]. Additionally, Casp12 may mediate cell apoptosis specifically activated by endoplasmic reticulum (ER) stress, including disruption of ER calcium homeostasis and accumulation of excess proteins in ER [11–13]. Casp12 exists in both truncated and full-length alleles in humans and as a full-length caspase in rodents [14]. Under certain pathologic conditions, Casp12 is expressed in human tissues, including proximal tubule cells of kidney [15] and cancers such as Hep-J5 cells [16, 17], multiple myeloma [18], gastrointestinal stromal tumor [19], glioblastoma [12] and NPC cells [13], suggesting a selective advantage in cancer cells [20]. However, the physiological function of Casp12 in cancer cells is still unclear.

Emerging evidence indicates an important contribution of caspases as regulators of nuclear factor-κB (NF-κB) signaling which activated in inflammation and cancer [21]. NF-κB activation induces the formation of pro-inflammatory cytokines in multiple cell types, such as macrophages, T cells and epithelial cells [22]. The main function of IκBα proteins is to retain NF-κB in the cytoplasm by masking its nuclear localization signal [21]. A variety of stimuli trigger transduction pathways resulting in phosphorylation of IκBα at Ser32 and Ser36 which leads to the proteasome-dependent degradation of IκBα [21]. Moreover, IκBα is cleaved by caspases to produce an amino-terminal truncated IκBα [23, 24]. The resulting truncated IκBα is degraded by the N-end rule pathway, thereby releasing and nuclear translocation of active NF-κB [24]. Activation of NF-κB may be able to induce the transcription factors, such as Slug, Snail, and Twist, resulting in the activation of MMP-2 and MMP-9 [25]. MMPs may exert their role in extracellular matrix turnover associated with cancer cell invasion [26, 27].

In this study, we investigated the effect of Casp12 on MMP-9-mediated cell invasion after treatment with phorbol-12-myristate-13-acetate (PMA), a potent activator of tumor cell invasion, in NPC cells [28]. We found that Casp12 induced the basal activity NF-κB via the post-translational degradation of IκBα. Moreover, up-regulation of Casp12 enhanced the effect of NF-κB-induced MMP-9 expression and accelerated cell invasion. This study suggested a role of Casp12 functioned as an important link between inflammatory and aggressive invasion in NPC cells.

## RESULTS

### Over-expression of Casp12 degraded IκBα and activated NF-κB

**Figure 1:**
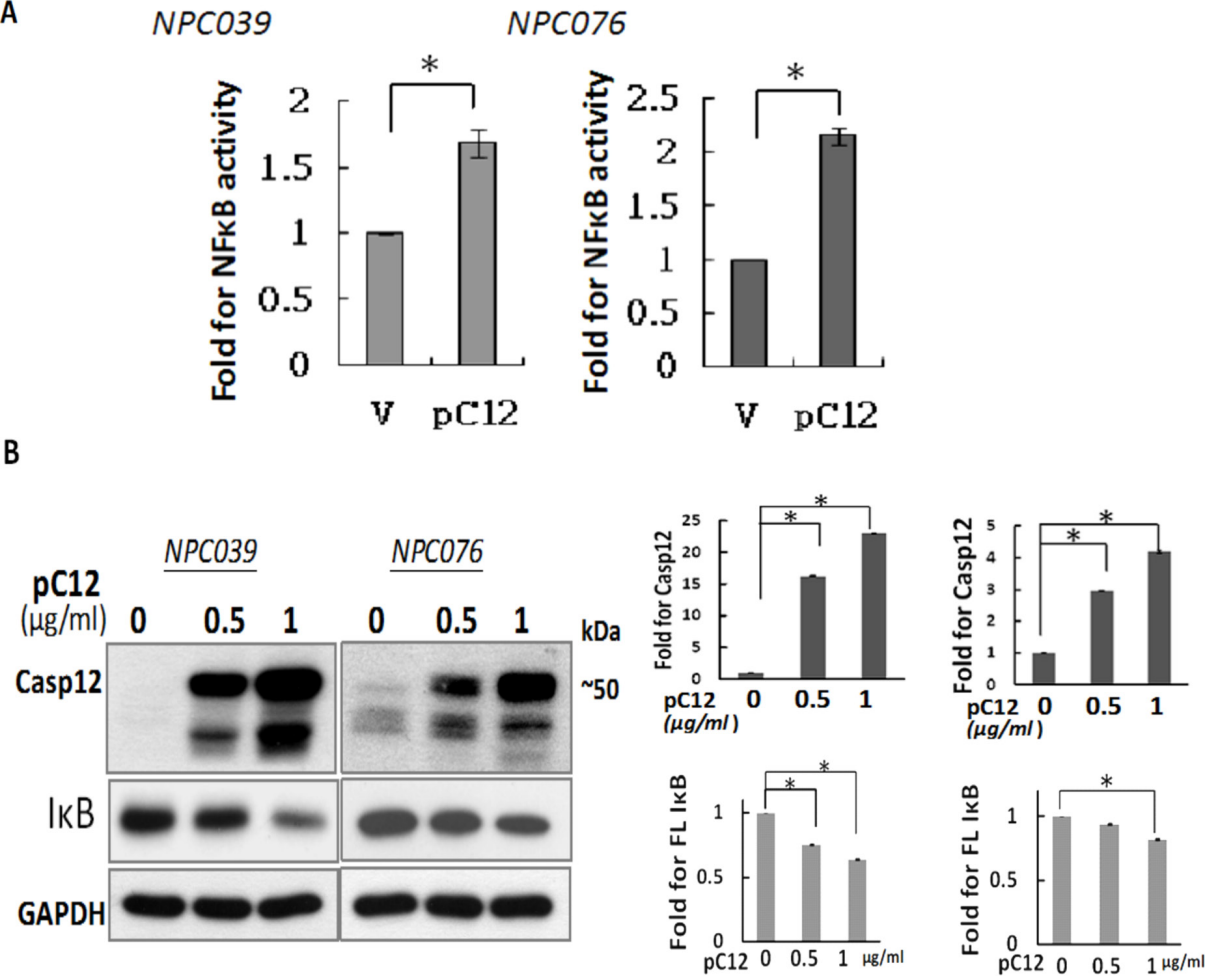
Over-expression of Casp12 increased NF-κB activity and degraded IκBα protein **(A)** Over-expression of Casp12 increased NF-κB activity. NPC cells were co-transfected with pcDNA-Casp12 (0.5 μg/ml) and NF-κB reporter plasmid in the presence of pSV-β-galactosidase vectors for 24 h. Luciferase activity is presented relative to the control group. **(B)** Ectopic expression of Casp12 induced the degradation of IκBα. NPC cells were transfected with pcDNA-Casp12 (pC12, 0.5~1 μg/ml) for 24 h and the cell lysates underwent western blot analysis with indicated antibodies. Data are mean±SD, N=3, *p<0.05.

NF-κB is a critical transcription factor involved in the regulated expression of inflammatory-related gene. We investigated the potential effect of Casp12 on NF-κB activation. NPC cells were co-transfected with pcDNA-Casp 12 (pC12) and NF-κB reporter plasmid for 24 h. Luciferase activity was significantly increased with Casp12 over-expression (Figure 1A). The possibility of Casp12 on degradation of IκBα was examined. NPC cells were transfected with pC12 in concentrations of 0.5 ~ 1 μg/ml for 24 h, and examined for Casp12-degraded IκBα expression. Over-expression of Casp12 dose-dependently decreased IκBα expression in NPC cells (Figure 1B). Thus, Casp12 could activate NF-κB pathway via degradation of IκBα.

### Casp12 mediated PMA-induced cell invasion

**Figure 2:**
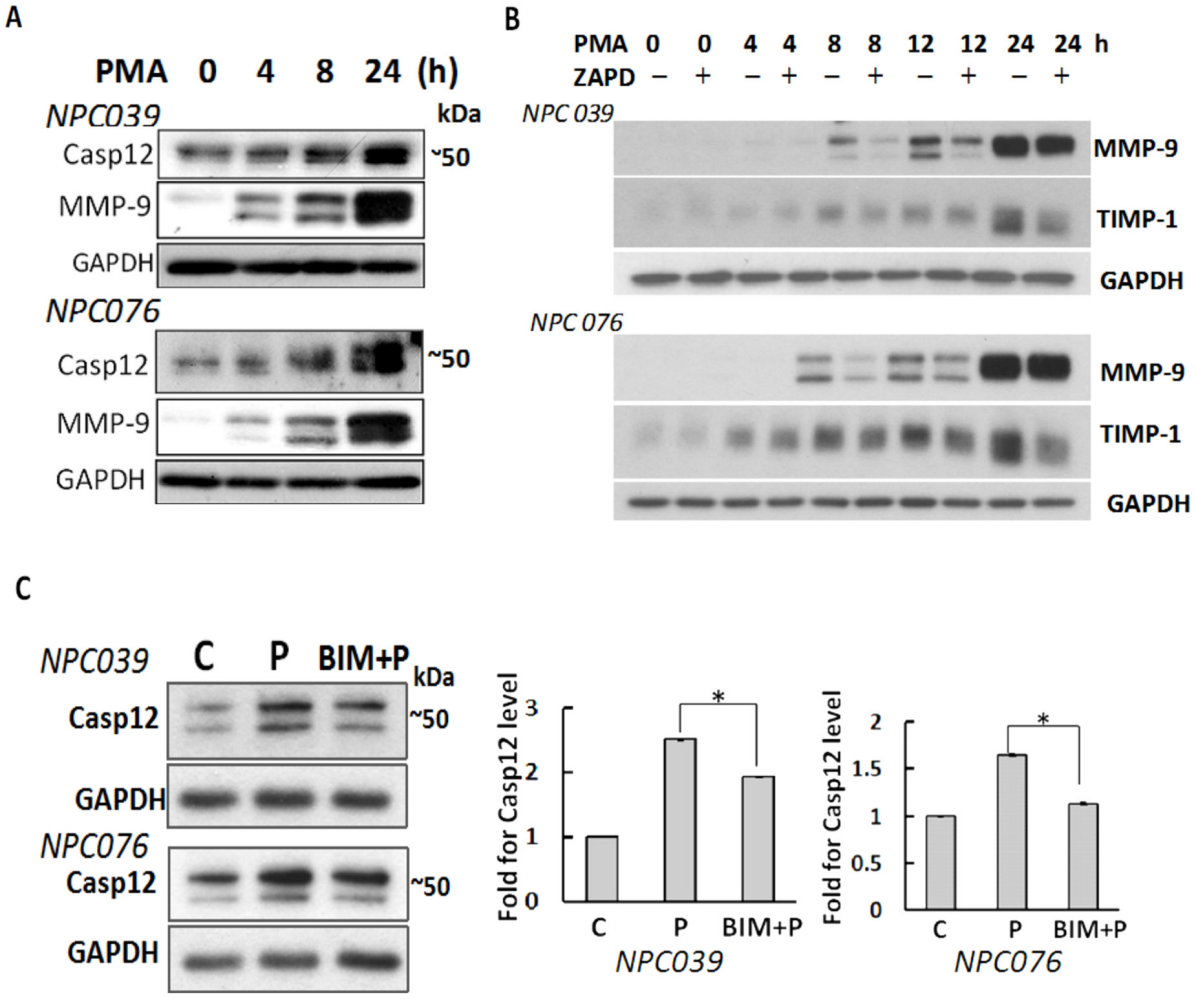
Up-regulation of Casp12 mediated PMA-mediated MMP-9 **(A)** PMA up-regulated Casp12 expression. NPC cells were treated with PMA as indicated time and cell lysates underwent western blot analysis with indicated antibodies (Casp12, MMP-9 and GAPDH). **(B)** Blockade of Casp12 decreased PMA-induced MMP-9 and TIMP-1 expressions. NPC cells were pre-treated with 20 μM Z-ATAD-fmk for 2 h, then exposed to PMA for indicated time. Cell lysates underwent western blot analysis with indicated antibodies. C. PMA induced Casp12 expression was PKC-dependent. NPC cells were pre-treated with BIM for 2 h, then exposed to PMA for 24 h. Cell lysates underwent western blot analysis with the indicated antibodies. **(C)** control group, P: PMA-treated group, The ratio of Casp12 to GAPDH was calculated. Data are mean±SD, N=3, *p<0.05.

PMA induces NPC cell invasion via up-regulation of MMP-9 [28]. We investigated the involvement of Casp12 in NPC cell invasion, NPC cells were treated with PMA for various times. PMA time-dependently up-regulated Casp12 and MMP-9 (Figure 2A). Treatment with Z-ATAD-fmk (a synthetic peptide that irreversibly inhibits Casp12) significantly inhibited the expressions of PMA-induced MMP-9 and tissue inhibitor of metalloproteinases-1 (TIMP-1), a binding partner of MMP-9, in a temporal manner (Figure 2B), suggesting Casp12 was implicated in the induction of MMP-9. PMA-mediated Casp12 expression was inhibited by 2-h pre-treatment with bisindolylmaleimide (BIM, a broad-spectrum PKC inhibitor), so the process was PKC-inducible.

**Figure 3:**
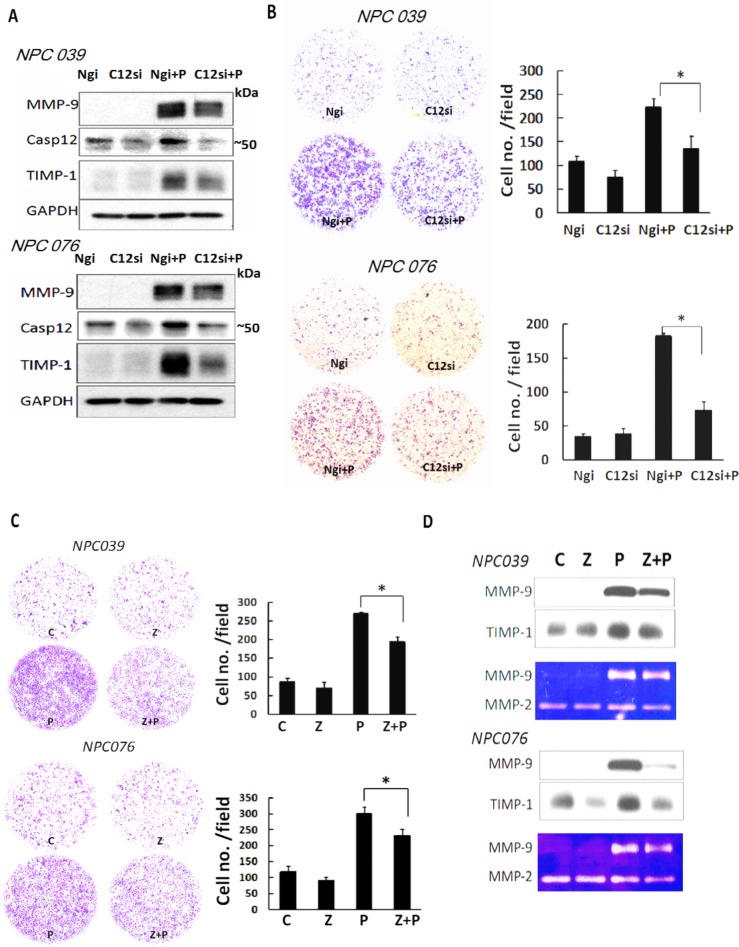
Depletion of Casp12 ameliorated PMA-mediated MMP-9 expression and cell invasion **(A)** SiRNA silencing of Casp12 expression decreased PMA-mediated cell invasion and MMP-9. NPC cells were transfected with Casp12 siRNA for 24 h and the transfected cells were exposed to PMA (100 nM) for 24 h. Cell lysates underwent western blot analysis with the indicated antibodies. **(B)** SiRNA silencing of Casp12 decreased PMA-mediated cell invasion. The transfected cells (1×10^5^) were seeded into the inner well of the Boyden chamber, pre-coated with matrix-gel and 10% FBS with or without 100 nM PMA introduced into the outer well of Boyden chamber for 24h. Cells that invaded to the lower surface of the filter were fixed, stained, photographed and counted. Representative plots of matrigel invasion assay are shown. Data are mean±SD, N=3, *p<0.05. **(C)** Inhibition of Casp12 activity decreased the level of PMA-induced MMP-9 and cell invasion. NPC cells were pretreated with 20 μM Z-ATAD-fmk, then co-incubated with PMA for 24 h. The cell invasion was detected by Boyden chamber assay. **(D)** Conditioned media from NPC cells treated with Casp12 inhibitor underwent to western blot analysis and gelatin zymography assay to detect MMP-9 and TIMP-1 expressions. Z: Z-ATAD-fmk. Data are mean±SD, N=3, *p<0.05.

To independently validate the specificity of Casp12 in cell invasion, we transfected NPC cells with Casp12 siRNA for 24 h, then the transfected cells were exposed to PMA for 16 h. SiRNA knockdown of Casp12 efficiently decreased Casp12 expression and markedly abolished PMA-induced MMP-9 and TIMP-1 expressions (Figure 3A). SiRNA knockdown of Casp12 significantly decreased PMA-induced cell invasion (Figure 3B). Z-ATAD-fmk treatment also abrogated PMA-mediated cell invasion approximately ~25% and ~23% in NPC 039 and NPC076 cells, respectively (Figure 3C). MMP-9 and TIMP-1 in conditioned media collected from Z-ATAD-fmk-treated cells consistently exhibited a similar effect, as investigated by western blot and gelatin zymography(Figure 3D). Thus, Casp12 may have a role in the modulation of PMA-induced MMP-9 expression.

### Casp12 mediated MMP-9 gene expression

**Figure 4:**
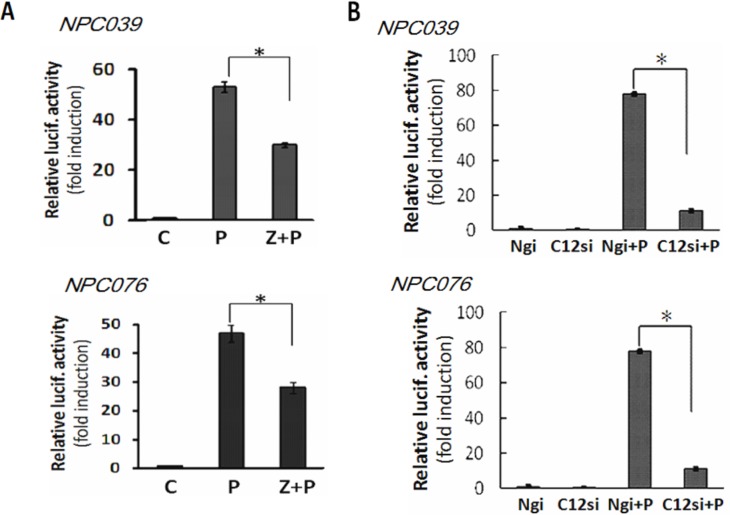
Casp12 mediated MMP-9 gene expression **(A)** Blockade of Casp12 activity decreased PMA-mediated reporter activity of MMP-9. NPC cells were transfected with pGL3-pMMP9-luc plasmid and pSV-β-galactosidase vectors for 24 h. The transfected cells were pre-treated with Z-ATAD-fmk for 2 h and then exposed to PMA for 24 h, then cell lysates were used to detect the luciferase activity. **(B)** SiRNA knockdown of Casp12 abrogated the reporter activity of MMP-9. NPC cells were cotransfected with Casp12 siRNA and pGL3-pMMP9-luc plasmid in the presence of pSV-β-galactosidase vectors for 24 h. The transfected cells were exposed to PMA for 16 h, then cell lysates underwent the luciferase activity assay. Luciferase activity is presented relative to the control group. Data are mean±SD, N=3, *p<0.05.

We investigated the possible signaling of Casp12 in regulating MMP-9. NPC cells were co-transfected with pGL-MMP9-Luc (a MMP-9 luciferase reporter plasmid) and Casp12 siRNA for 24 h, then the transfected cells were exposed to PMA for 16 h. SiRNA knockdown of Casp12 significantly decreased PMA-induced reporter activity of MMP-9 (Figure 4A). Treatment with Z-ATAD-fmk also markedly decreased PMA-mediated reporter activity for MMP-9 (Figure 4B). Thus, Casp12 may regulate MMP-9 gene expression at the transcriptional level.

### Casp12 mediated the activation of NF-κB

**Figure 5:**
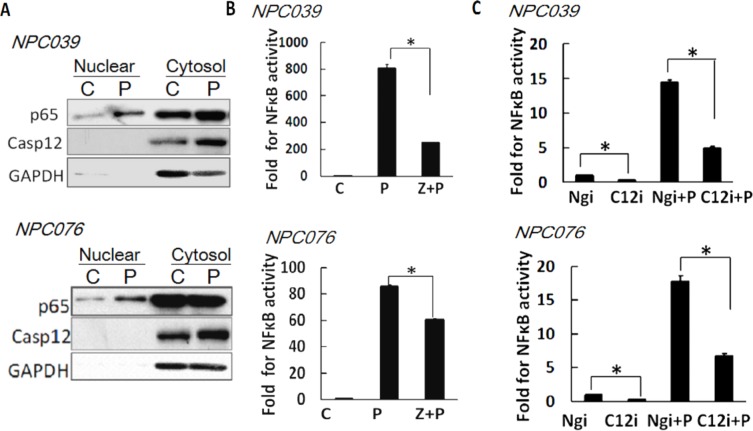
Casp12 was involved in the modulation of NF-κB activity **(A)** PMA induced the p65 nuclear distribution. NPC cells were exposed to PMA for 24 h and then the cytoplasmic fraction and nuclear fraction underwent western blot analysis with indicated antibodies (p65 and Casp12). GAPDH was shown as the cytoplasmic loading control. **(B)** Treatment with Casp12 inhibitor abrogated PMAinduced NF-κB activity. NPC cells were transfected with NF-κB reporter plasmids and pSV-β-galactosidase vector for 24 h. The transfected cells were pre-treated with Z-ATAD-fmk for 2 h and co-incubated with PMA for 24 h, then cell lysates were used to detect the luciferase activity. **(C)** SiRNA knockdown of Casp12 abrogates the NF-κB activity. NPC cells were co-transfected with Casp12 siRNA and NF-κB reporter plasmid in the presence of pSV-β-galactosidase vectors for 24 h. The transfected cells were exposed to PMA for 24 h. Luciferase activity is presented relative to the control group. Data are mean±SD, N=3, *p<0.05.

NF-κB plays a predominant role in *MMP*-*9* gene induction [21]. We examined the possible contribution of Casp12 on NF-κB activation. PMA induced the nuclear translocation of p65 (NF-κB) and increased Casp12 expression distributed in cytoplasmic fraction (Figure 5A). Next, we transfected NPC cells with NF-κB reporter plasmid for 24 h, then the transfected cells were co-incubated with PMA and Z-ATAD-fmk for 16 h. Z-ATAD-fmk significantly inhibited the luciferase activity of NF-κB induced by PMA (Figure 5B). Next, we co-transfected NPC cells with Casp12 siRNA and NF-κB reporter plasmid for 24 h, and then the transfected cells treated with PMA for 16 h. SiRNA knockdown of Casp12 significantly decreased the luciferase activity of NF-κB and markedly attenuated PMA-induced NF-κB reporter activity (Figure 5C). Thus, a functional role of Casp12 was on modulation of NF-κB activity.

### Casp12 induced the degradation of IκB protein

**Figure 6:**
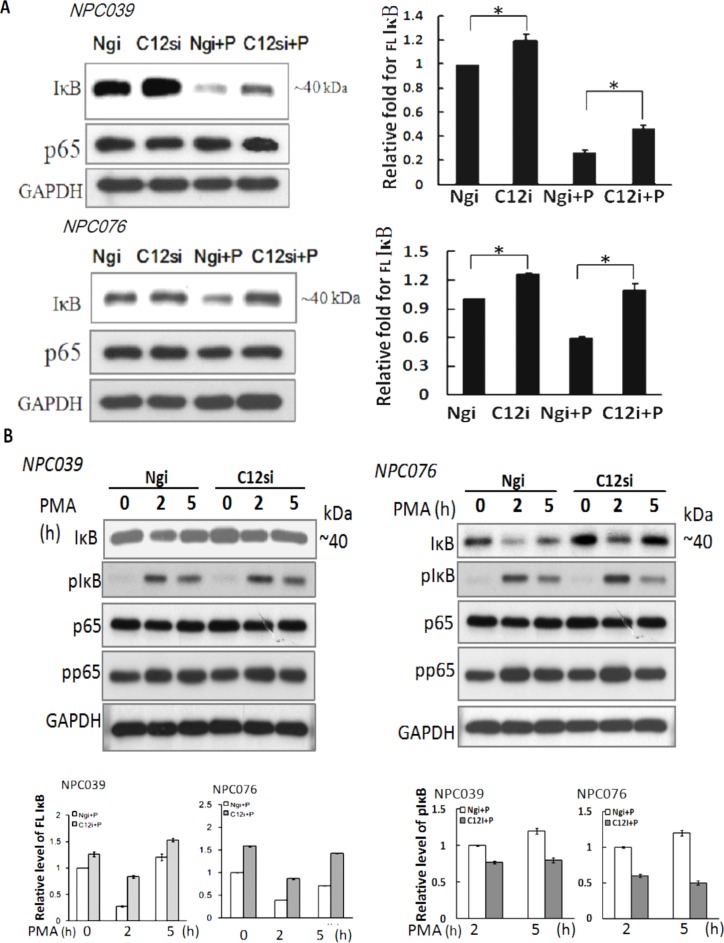
SiRNA knockdown of Casp12 increased IκBα expression **(A)** SiRNA knockdown increased the basal IκBα expression. NPC cells were transfected with Casp12 siRNA for 24 h, then exposed to PMA for 24 h. Cell lysates underwent immunoblotting for indicated antibodies (IκBα, p65 and GAPDH). **(B)** SiRNA knockdown of Casp12 reversed the PMA-degraded IκBα expression. NPC cells were transfected with Casp12 siRNA for 24 h, then exposed to PMA for the indicated time. Cell lysates underwent western blot analysis with indicated antibodies (IκBα, p-IκBα, p65 and p-p65). Data are mean±SD, N=3, *p<0.05.

The effect mechanism of Casp12 on NF-κB activation warrants further investigation. Degradation of IκBα is a decisive step in activation of NF-κB. We investigated whether Casp12 had any effect on IκBα and p65 expressions. We transfected NPC cells with Casp12 siRNA for 24 h, then the transfected cells were exposed to PMA for 24 h. SiRNA knockdown of Casp12 significantly increased IκBα expression and markedly reversed PMA-induced IκBα degradation, but did not affect p65 expression (Figure 6A). The results indicated significant Casp12-dependence in modulating the expression of IκBα in NPC cells.

Activation of NF-κB mainly occurs via phoshorylation of inhibitory molecules, including IκBα. We investigated the effect of Casp12 on phosphorylation of IκBα or p65 (p-IκBα or p-p65). NPC cells were transfected with Casp12 siRNA for 24 h and then transfected cells were exposed to PMA in a various time. At 2-h time point of PMA treatment, the protein IκBα dropped sharply in level associated with markedly increased p-IκBα expression in Ngi-transfected cells (Figure 6B). At 5-h time point of PMA treatment, IκBα expression, but not p-IκBα, was higher than at 2-h time point. PMA treatment did not affect p65 expression, but increased p-p65 expression at 2-h time point in Ngi-transfected cells. The results suggested the role of p-IκBα on IκBα degradation at the early phase of PMA treatment. Consistent with the result of Figure 5A, transfection with Casp12 siRNA also increased the basal level of IκBα expression, but did not affect p65 expression (Figure 6B). Importantly, target silencing of Casp12 siRNA abolished PMA-mediated degradation of IκBα, but did not change PMA-mediated p-p65 and p-IκBα expressions. The results indicated that PMA-degraded IκBα expression not only induced through the phosphorylation pathway, but also induced via the presence of Casp12 in NPC cells.

### PMA increased the transcripts of IκBα

**Figure 7:**
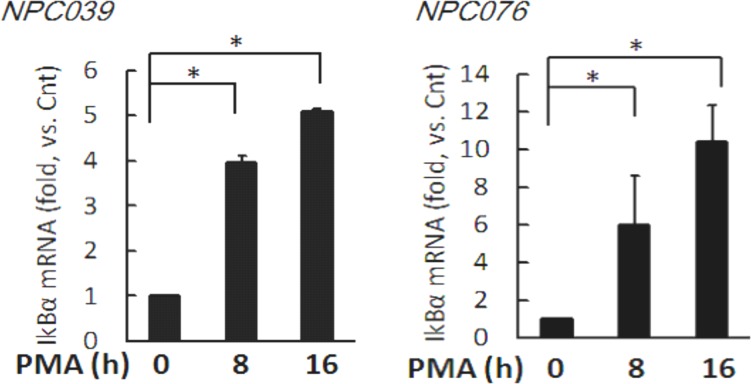
PMA time-dependently increased the transcript of IκBα NPC cells were treated with 100 nM PMA for the indicated time. The mRNAs expressions of IκBα and GAPDH were determined by q-RTPCR. Data are mean±SD, N=3, *p<0.05.

We investigated the effect of PMA on the gene expression of IκBα. NPC cells were exposed to PMA for indicated time and the transcripts were assessed by q-RTPCR. Significantly, PMA time-dependently increased IκBα mRNA expression by 3.97 ± 0.16, and 5.1 ± 0.05 fold and 5.96 ± 2.65 and 10.40 ± 1.98 fold at 8-h and 16-h time points in NPC039 cells and NPC076 cells, respectively (Figure 7).

### Casp12 mediated the post-translational degradation of IκBα

**Figure 8:**
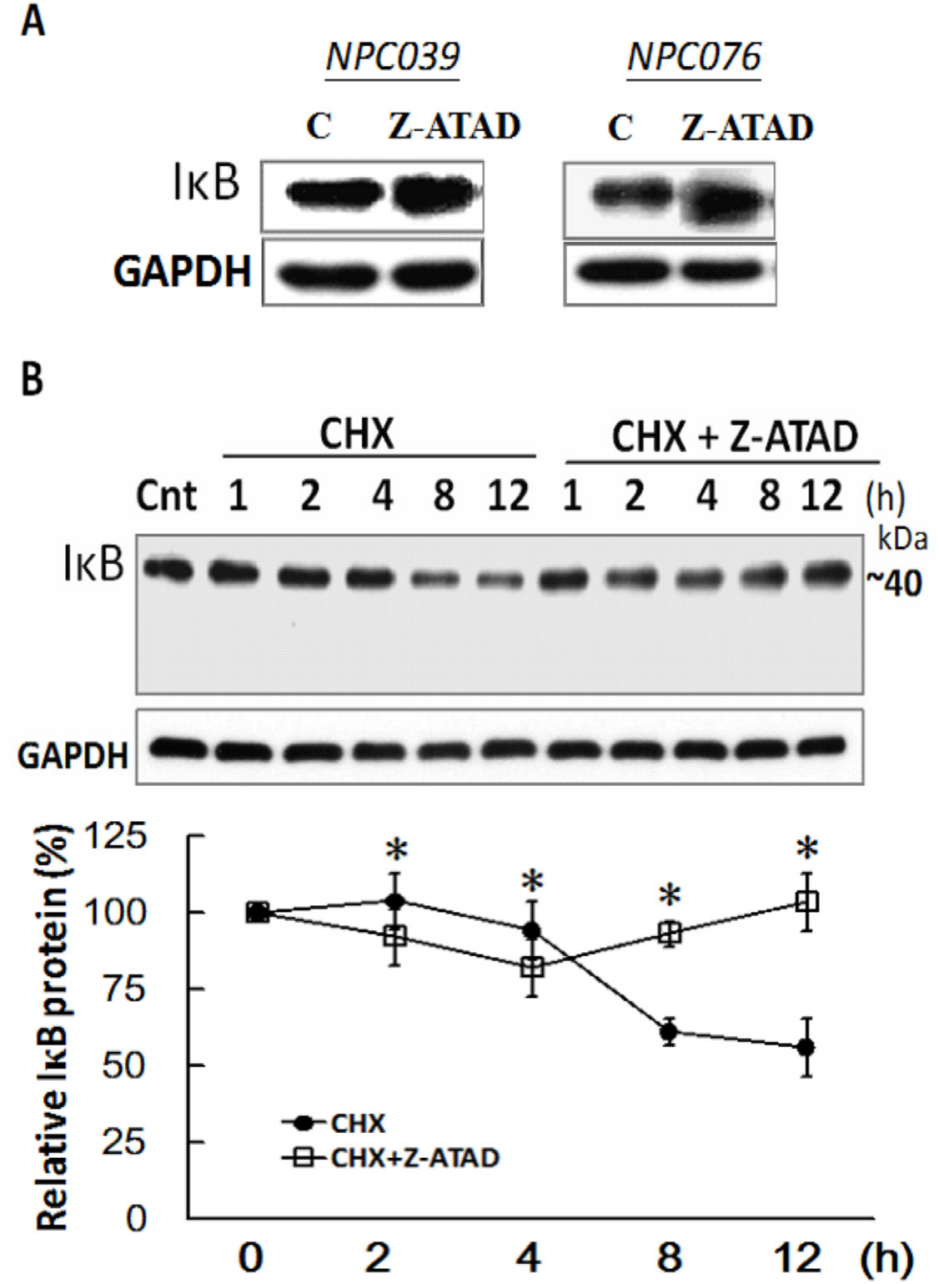
IκBα was post-translational degradation mediated by Casp12 **(A)** Suppression of basal Casp12 activity increased the basal IκBα expression. NPC cells were treated with Z-ATAD-fmk for 24 h, and the cell lysates underwent immunoblotting with indicated antibodies. **(B)** NPC cells were treated with CHX for the indicated time in the presence of Z-ATAD-fmk and the cell lysates underwent immunoblotting with indicated antibodies. Data are mean±SD, N=3, *p<0.05 (CHX vs. CHX+Z-ATAD).

We investigated the basal activity of Casp12 involved in regulating the IκBα expression. NPC cells were treated with Z-ATAD-fmk for 24 h and the IκBα expression was examined. Markedly, Z-ATAD-fmk treatment increased IκBα expression in NPC cells (Figure 8A). We examined the possibility of Casp12 on the post-translational degradation of IκBα, NPC cells were treated with cycloheximide (CHX) in the presence/absence of Z-ATAD-fmk for the indicated time. Addition of CHX to NPC cells significantly decreased IκBα expression by 61.3 % and 56.2 % at 8- and 12-h time points, respectively, which were significantly blocked in the presence of Z-ATAD-fmk (Figure 8B). The results might suggest the basal activity of Casp12 in the modulation of IκBα degradation in NPC cells.

## DISCUSSION

Casp12 has an anti-inflammatory function during infection [29], which expressed in cancer cells implies the simultaneous presence of some selective benefit for cancer pathogenesis [20]. In this study, we expanded a new function and novel regulatory mechanism of Casp12 implicated in NPC cell invasion. We suggested that Casp12 may activate NF-κB by targeting the post-translational degradation of IκBα. The alternative activated pathway of NF-κB is induced by Casp12 might be implicated in MMP-9-mediated carcinoma cell invasion. Thus, a signaling of Casp12/NF-κB may provide an inflammatory regulatory response to determine NPC cell fate.

The cysteine-aspartic protease Casp12 is largely known for their role in controlling cell death and inflammation [30]. In response to endoplamic reticular stress, Casp12 is involved in induction of Casp3-mediated apoptosis pathway in NPC cells [13]. This study indicated that up-regulation of Casp12 was protein kinase C (PKC)-inducible (Figure 2) and over-expression of Casp12 was implicated in activation of NF-κB protein and degradation of IκBα (Figure 1). Treatment of NPC cells with PMA promoted NPC cell invasion and accompanied with increased MMP-9 expression [28]. In contrast, suppression of Casp12 markedly decreased the PMA-mediated cell invasion and MMP-9 expression (Figure 3 & Figure 4). The involvement ofCasp12 in MMP-9 expression was further validated on the transcriptional regulation of MMP-9 (Figure 5). Aberrant NF-κB activity is a hallmark of cancer and chronic inflammatory diseases. Mechanistically, Casp12 binds to Rip2, displacing Traf6 from the signaling complex, inhibiting its ubiquitin ligase activity, and blunting NF-κB activation [31]. By contrast, this study ascertained that a basic mechanistic effect of Casp12 governing the activation of NF-κB. Suppression of Casp12 effectively attenuated the basal activity of NF-κB and the PMA-induced NF-κB reporter activity (Figure 5B & 5C). Thus, induction of Casp12/NF-κB signaling might be associated with MMP-9-induced aggressive metastasis in carcinoma cell.

*Degradation of* IκBα is a seminal step in *activation of NF*-*κB*. It is conceivable that an additional signal is needed for effective IκBα cleavage by caspase and subsequent degradation of the cleaved IκBα by the proteasome in drosophila and mammalian cells [24, 32]. IκBα can be cleaved by casp8 that activates the NF-κB pathway [33]. In this study, the basal level of IκBα expression was markedly increased after suppression of Casp12 with Z-APAD or its siRNA (Figure 8A & Figure 5). Furthermore, the effect of Casp12 on the degradation of IκBα was verified in a post-translational level (Figure 8B). Thus, activation of NF-κB signaling could partly rely on the effect of Casp12 that provided a contribution to the invasiveness in NPC cells.

Casp12 occurs primarily in individuals of African descent and is linked with susceptibility to sepsis. The function of Casp12 identified thus far would suggest a rather anti-inflammatory role [34], although it is revealed as a negative regulator of inflammasomes [15]. This study indicated the potential effect of Casp12 was on the activation of NF-κB by the degradation of IκBα in NPC cells. The importance of Casp12 in hardwiring NF-κB signal transduction pathways to the regulation of MMP-9 gene may be a therapeutic target for carcinoma. This finding may provide a possible link between chronic inflammation and cancer metastasis.

## MATERIALS AND METHODS

### Cell culture & reagents

Human nasopharyngeal carcinoma cell lines NPC076, and NPC039 are isolated from nasopharyngeal squamous cell carcinoma [35]. The cells are maintained in basal medium (DMEM/F-12 at 1:1, v/v; Invitrogen, Carlsbad, CA) supplemented with 5% fetal bovine serum in a humidified incubator at 37°C under 5% CO_2_/95% air. The Z-ATAD-fmk is a specific inhibitor for Casp12 purchased from BioVision or ProteinTec. The antibody for IκBα was purchased from Santa Cruz (SC-371). Other antibodies or reagents were purchased from Cell Signaling, ABcam, or Sigma Aldrich. A plasmid for cDNA-Caspase 12 (pC12) was a gift from Junying Yuan (Addgene plasmid # 35574) [11].

### SiRNA transfection

Specific small interfering RNAs (siRNAs) were used to silence Casp12 expression. SiRNA targeting parts of Casp12 mRNA was purchased from Santa Cruz Inc. (sc-72797, siRNA products consist of pools of three target-specific 19-25 nt siRNAs). Negative control siRNA (Ngi) was as previously described [13]. The transfection was performed using Lipofectamine 2000 according to the manufacturer's protocol. The effectiveness of siRNA silencing was assayed by western blot analysis.

### Transient transfection and luciferase reporter assays

Transient transfection of the luciferase reporter plasmids involved use of Lipofectamine 2000 (Invitrogen). NPC cells were transfected with pMMP-9-RE luciferase reporter plasmid or NF-κB reporter (Luc) (pGL4.32[*luc2P*/NF-κB-RE/Hygro] vector contains five copies of an NF-κB response element (NF-κB-RE) and pSV-β-galactosidase control vector (Promega) for 24 h. The transfected cells were incubated with PMA with or without significantly for indicated times and the lysates were used directly for luciferase activity assay (Promega). The β-Galactosidase enzyme assay (Promega) involved use of the same lysates to standardize the transcription efficiency. The luciferase activity of induction was detected using the Luciferase Assay System (Promega).

### Protein lysates and western blot analysis

Before exposure to PMA, NPC cells were treated with Casp12 siRNA, pharmaclogical inhibitors or transfected with ectopic Casp12. Cellular lysates were prepared by Mammalian Protein Extraction Reagent (M-PER; Pierce Chemical Co.). Cytosolic and nuclear extracts were prepared by using the NE-PER Nuclear and Cytoplasmic Extraction Reagents kit (Thermo Fisher Scientific). The cellular and subcellular extracts underwent SDS-PAGE and blotted onto polyvinylidene difluoride membranes (Immobilon(TM)-P, Millipore). Blots were probed with primary antibodies then appropriate horseradish peroxidase-conjugated secondary antibodies. Immunoreactive protein bands were developed by Enhanced Chemiluminescence (ECL) (Perkin Elmer LAS Inc.).

### Cell invasion assays

The Boyden chamber cell invasion assay involved use of transwell chambers with 8-μm pore size membranes and 6.5 mm diameter (Becton–Dickinson). NPC Cells were added to matrix gel-precoated upper chamber at 1 × 10^5^ cells per well. The lower chamber was filled with DMEM containing 10% FBS with or without 100 nM PMA. After 24 h, the cells that migrated to the lower side were fixed in 4 % paraformaldehyde, stained with 0.25 % crystal violet, and counted under a light microscope in five predetermined fields.

### MMP-9 zymography assay

NPC cells were incubated PMA in the presence/absence of Z-ATAM-fmk for a given time, then the conditioned media (CM) were collected. CM was separated by 10% SDS-PAGE containing 0.1 % gelatin. After electrophoresis, gels were washed twice in washing buffer (2.5 % Triton X-100 in dH2O) at room temperature for 30 min each time to remove SDS, then incubated in reaction buffer (10 mM CaCl_2_, 0.01 % NaN_3_ and 40 mM Tris-HCl, pH 8.0) at 37°C for 12 h to allow proteolysis of the gelatin substrate. Bands corresponding to the activity were visualized by negative staining using Coomassie Brilliant blue R-250 (Bio-Rad Laboratories, Richmond, CA) and molecular weights were estimated by referencing prestained SDS-PAGE markers.

### qRT-PCR analysis

Total RNA was extracted from cells using TRIzol Reagent (Invitrogen). The amount of RNA was measured by a NanoDrop spectrophotometer (Thermo Scientific). Reverse transcription (RT) was performed with 1 μg total RNA for complementary DNA synthesis using an iScript cDNA Synthesis Kit (Bio-Rad). Quantitative real-time PCR (qPCR) was performed using the iQ SYBR Green Supermix kit (Bio-Rad) and monitored on a LightCycler 480 system (Roche). The qPCR conditions were as follows: 3 min at 95°C followed by 40 cycles of 10 sec at 95°C and 30 sec at 55°C. The specific primers used were as follows: IkBα-F: 5′-GAAGCCGCTGACCATGGAA-3′ 5′-GATCACAGCCAAGTGGAGTGGA -3′; GAPDH-F: 5′-CACCCACTCCTCCACCTTTG-3′; and GAPDH-R: 5′-CCACCACCCTGTTGTTGTAG-3′. Relative mRNA levels were normalized to GAPDH mRNA levels using the 2-ΔΔCt method [36].

### Statistical analysis

Data were presented as mean ± SD. Statistical significance between groups was assessed by unpaired student's *t*-test. A p value of less than 0.05 was considered to be significant.
